# The effects of radiation on myeloid lineage immune cells within the rodent urinary bladder: a systematic review

**DOI:** 10.1007/s11255-023-03748-1

**Published:** 2023-08-24

**Authors:** Jessica Smith, Rimaz Toto, Christian Moro

**Affiliations:** Faculty of Health Sciences and Medicine, Bond University, Queensland, 4226 Australia

**Keywords:** Radiation-induced cystitis, Haematopoietic stem cell transplantation, Radiotherapy, Cystitis, Urinary bladder, Immune cells

## Abstract

**Purpose:**

Radiotherapy is a prominent therapy for many malignant and non-malignant disorders, though it can cause side effects such as radiation-induced cystitis. Current research has highlighted a role for mast cells and macrophages in the prognosis of such radiation-induced toxicities. However, the prognostic value of these immune cells in the pathophysiology of radiation-induced cystitis is not clear. As such, a systematic review was conducted to assess myeloid-lineage immune cells for their prognostic value in radiation-induced cystitis to address this gap in literature.

**Methods:**

The protocol was registered in PROSPERO, and searches were performed in PubMed, Embase and Web of Science databases for pre-clinical rodent studies on radiation-induced cystitis.

**Results:**

After de-duplication, 153 articles were screened for relevancy by title and abstract. Title and abstract screening deemed 64 studies irrelevant. The remaining 85 studies were full-text screened, yielding seven unique articles for data extraction. Most included studies had an unclear risk of bias. The findings of this systematic review suggest that the prognostic value of myeloid-lineage immune cells in radiation-induced cystitis is still unclear, indicating a need for further research in this field.

**Conclusion:**

Although the studies reviewed provide some insight into the role of these immune cells in disease pathology, the limited number of studies and unclear risk of bias further highlights a need for additional, high-quality research in this area. In summary, this systematic review highlights a need to understand the involvement of immune cells in radiation-induced cystitis pathophysiology and lay the groundwork for further research in this area.

**Trial registration:**

PROSPERO registration: CRD42022345960

## Introduction

Radiation-induced cystitis is an inflammatory condition that can develop as a side-effect of abdominopelvic radiation exposure [1]. The incidence of radiation-induced cystitis is suggested to range from 23% to 80%, with this variability likely being a result of variable doses of radiotherapy across medical subspecialties [1]. External-beam radiation therapy (EBRT) is a common type of radiation therapy, involving the use of a linear accelerator to deliver a focussed beam of radiation to malignant tissue. Radiation-induced cystitis can also be induced by myeloablative therapy, which is used a conditioning regiment for hematopoietic stem cell transplantation (HSCT) in the treatment of various hematological diseases by delivering high-dose radiation to the entire body [2].

Radiation-induced cystitis is thought to develop triphasically. The first phase of radiation-induced cystitis is characterized by acute inflammation, involving a loss of the urinary bladder's glycosaminoglycan and urothelium cell layers, infiltration of immune cells, vasodilation, and hyperplasia of bladder endothelium. The acute phase is then followed by a symptom-free phase, lasting from months to years [3, 4]. The third and final phase constitutes a chronic inflammatory response. The chronic phase is best characterized by collagen deposition, loss of smooth muscle epithelia, hemorrhaging, thinning of the urothelial wall and an influx of pro-inflammatory immune cells. However, the extent of immune cell distribution and prevalence during acute and chronic phases of radiation-induced cystitis has not been well established, and more importantly, whether these characteristics apply to myeloablative therapy considering its immunosuppressive nature. Though current research has highlighted a possible role for urinary bladder mast cells [3, 5] and macrophages [6] in the development of radiation-induced cystitis, their specific role is unclear.

In pre-clinical literature, mast cells have been associated with the development of fibrosis in various tissues in response to radiation exposure [7–11]. Specifically, mast cells are believed to release various chemokines, such as transforming growth factor-β (TGF-β), which promotes fibroblast recruitment and proliferation [12], causing fibrosis. Urinary bladder fibrosis has been previously associated with underactive bladder and impaired bladder compliance disorders [13, 14]. Clinical presentations of radiation-induced cystitis range from increased urinary frequency and urgency, dysuria, nocturia, increased risk of bladder infections, hematuria and suprapubic pain, as reviewed by Zwaans, Nicolai [15]. Mast cells are also believed to disrupt vasculature by increasing the permeability of blood vessels [16] as well as playing a direct role in chemical cascades that result in radiation-associated toxicities [9]. Similarly, macrophages are also suggested to play a role in the pathophysiology of radiation-induced cystitis, namely in promoting fibrosis and inflammation, as reviewed by Helissey, Cavallero [6]. Other myeloid-lineage immune cells, such as monocytes, neutrophils, eosinophils and basophils, may be involved, but less is known of their role in the development of radiation-induced inflammation. Hence, this systematic review aims to establish the potential prognostic value of myeloid-derived immune cells in radiation-induced cystitis by reviewing current pre-clinical literature on their involvement in tissue injury and repair of the irradiated urinary bladder.

## Materials and methods

This systematic review followed the Preferred Reporting Items for Systematic Reviews and Meta-Analyses statement (PRISMA) guidelines [17]. The protocol for the systematic review was prospectively developed and registered in PROSPERO (CRD42022345960).

### Search strategy

Embase, PubMed, and Web of Science online databases were searched for published articles from database inception to 16 November 2022. Language and year of publication restrictions were not imposed in the systematic review. Studies were identified using the following terms: radiation, radiotherapy, hematopoietic stem cell transplantation, urinary bladder, urothelium, cystitis, rodents, immunomodulation, fibrosis, inflammation, radiation effects, radiation fibrosis syndrome, leukocytes, macrophages, monocytes, mast cells, neutrophils, eosinophils and basophils. The search string used to search the PubMed database is found below:

(“Radiation, Ionizing”[tiab] OR “Radiation”[tiab] OR “Radiotherapy”[tiab] OR “Haematopoietic stem cell transplantation”[tiab]) AND (“Urinary Bladder”[tiab] OR “Urothelium”[tiab] OR “Cystitis”[tiab] OR “Bladder”[tiab]) AND (“Rodent”[tiab] OR “Mice”[tiab] OR “Rats”[tiab] OR “Guinea Pigs”[tiab]) AND (“Immunomodulation”[tiab] OR “Fibrosis”[tiab] OR “Inflammation”[tiab] OR “Radiation Effects”[tiab] OR “Radiation Exposure”[tiab] OR “Radiation Injuries”[tiab] OR “Radiation Fibrosis Syndrome”[tiab] OR “Leukocytes”[tiab] OR “Mast Cells”[tiab] OR “Neutrophils”[tiab] OR “Eosinophils”[tiab] OR “Basophils”[tiab] OR “Macrophages”[tiab] OR “Monocytes”[tiab]).

### Eligibility criteria

Studies were included in this systematic review if they satisfied the following criteria: (i) included radiotherapy, (ii) assessed myeloid-lineage immune cell function, prevalence or distribution, and (iii) evaluated fibrosis, inflammation or immunosuppression in irradiated rodent urinary bladders. Only original experimental animal studies were included. Animals with infectious diseases or studies without healthy control animals were excluded. Reviews, systematic reviews, meta-analyses and conference abstracts were excluded from this systematic review. Human studies were also excluded from this review but were included in the introduction and discussion to provide a contextual clinical application.

### Study screening, data extraction and quality assessment

Two independent reviewers performed study screening, and disagreements were resolved by the third reviewer. After duplicate removal, title and abstracts were screened to exclude irrelevant studies. One author retrieved full-text articles, and two authors screened full-text articles against the eligibility criteria for inclusion in this systematic review.

After full-text article screening, two reviewers extracted data from eligible studies. Information relating to the population (total animal numbers, animal species, strain, sex and age) and intervention (type of radiation, dosage, target tissue, adjuvant treatments) was extracted, in addition to information relating to primary and secondary outcome measures. The primary outcome measure was to assess the impact of radiation on myeloid-lineage immune cells in relation to their function, prevalence or distribution. The secondary outcome measure was to assess the impact of radiation on the urinary bladder with regard to inflammation, fibrosis and immunosuppression. Where possible, data relating to the mean, standard error of mean, or standard deviation were extracted from the main text. Only data from the last time point were extracted when outcome measures were assessed at multiple times. Estimates were cross-checked, and variances in data extraction exceeding 10% were resolved through discussion between all reviewers. Extracted data were then entered into Covidence by one reviewer and checked by another reviewer independently.

Two review authors independently assessed the risk of bias for each included study using the criteria outlined by Systematic Review Centre for Laboratory animal Experimentation [18]. All disagreements were resolved by discussion or by referring to a third author.

## Results

### Study selection and qualitative assessment

**Fig. 1:**
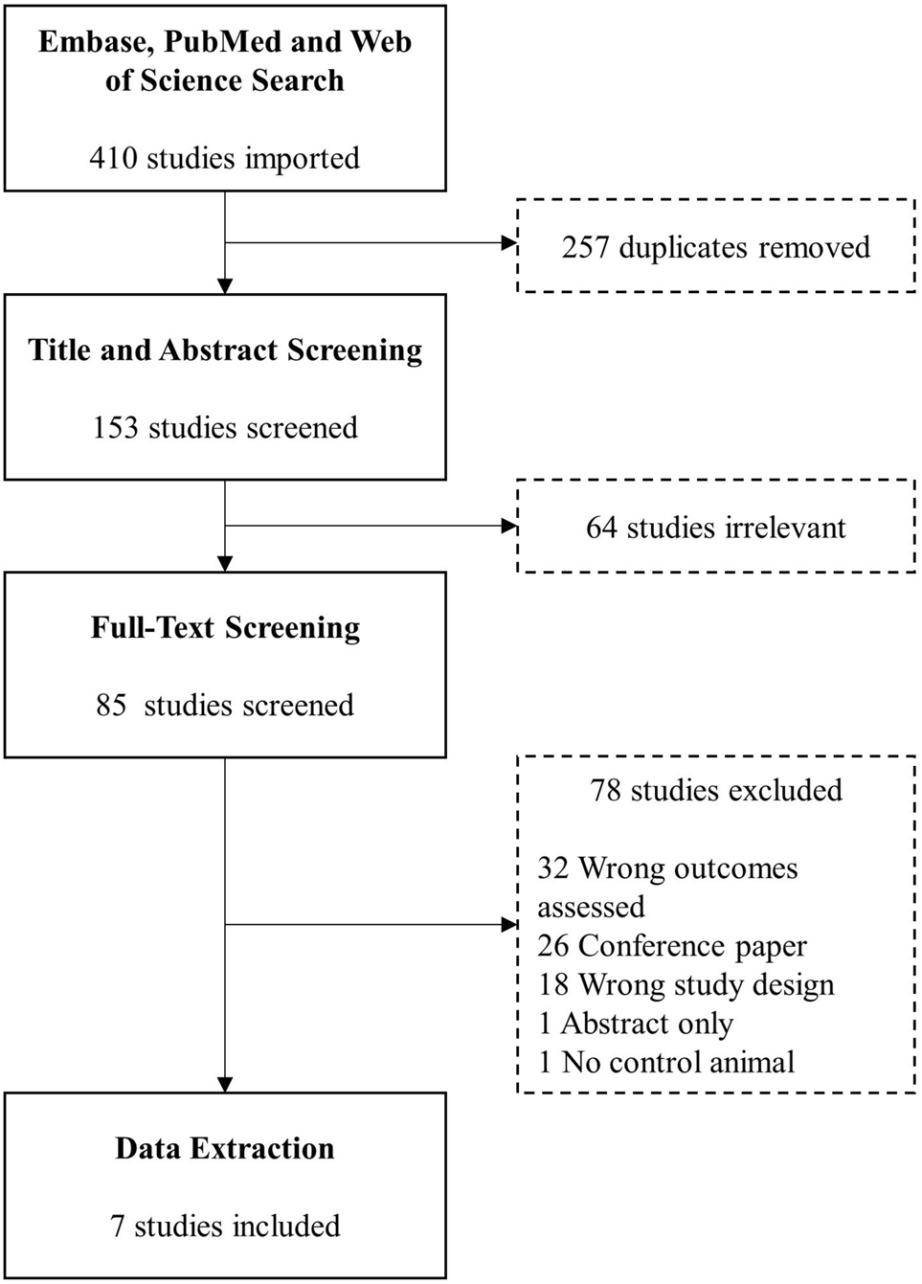
PRISMA search strategy flow chart

A total of 257 studies were identified in the initial online database search after duplicate removal. 153 studies’ titles and abstracts were then assessed for relevancy. From the title and abstract screening, 64 studies were deemed irrelevant. The remaining 85 studies were full text screened against the inclusion and exclusion criteria. A total of seven unique studies were included for data extraction (Fig. 1).

### Risk of bias assessment

**Fig. 2:**
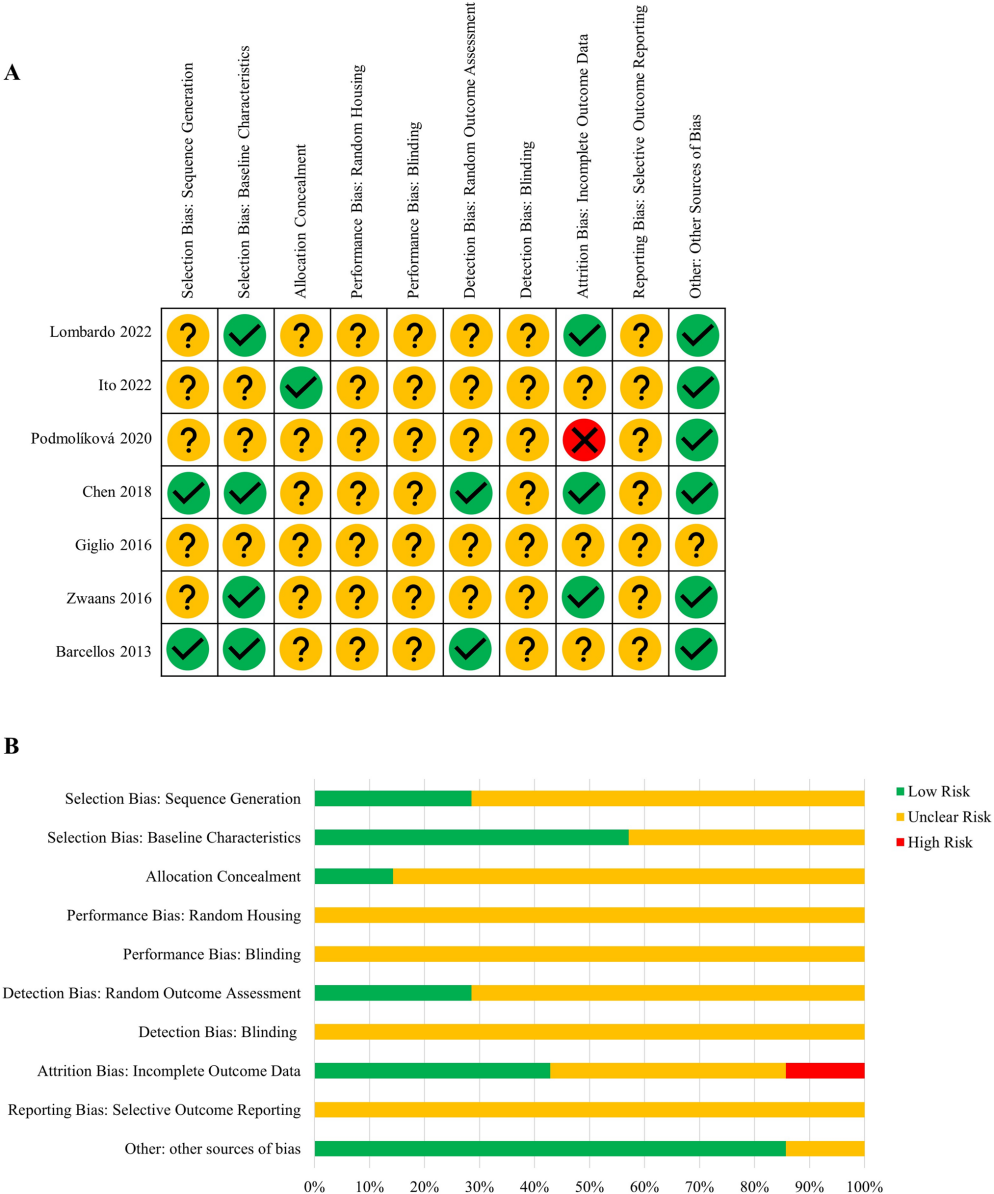
SYRCLE risk of bias assessment of included studies: **A** indicates the scoring of individual studies, and **B** indicates overall scores, presented as a percentage. Symbols: green tick indicates low risk, the yellow question mark indicates unclear risk, red cross indicates a high risk of bias

Study quality was assessed using the Systematic Review Centre for Laboratory animal Experimentation (SYRCLE) guidelines [18]. Most included studies had an unclear risk of bias, illustrated in Fig. 2. No studies stated randomized housing or blinding in performance and detection biases. One study did not explain variance in sample sizes [19], scoring high for risk of bias in “Attrition Bias: Incomplete Outcome Data.” All studies had an unclear risk of selective outcome reporting bias. All studies in “Other: Other Sources of Bias” included disclosure of ethics approval or potential conflict of interest statement except for Giglio, Wasén [20].

### Study characteristics

Of the seven included studies, five analyzed rat urinary bladders [19–23], and two analyzed the mouse urinary bladder [8, 24]. Five studies were conducted on female urinary bladders [8, 19–21, 24] and two studies were conducted on male urinary bladders [22, 23]. One author was contacted to clarify the age of rodents analyzed [20]. The most common rodent strain was Sprague–Dawley rats (Table 1).

**Table 1:** Study characteristics summary

Author	Country	Species (strain)	Age	Sex	Type of radiation	Dose	Primary outcomes	Secondary outcomes
Ito, Yamamoto [21]	Japan	Rats (Fischer F344)	9weeks	Female	X-irradiation, targeted at the pelvis, filtered through a 2 mm lead plate	40 Gy total dose in eight 5 Gy fractions (two times a week); 4.9 Gy/min dose rate at 150 kV and 20 mA for 1.021 min	Radiotherapy reduced the prevalence of CD68þ and CD163 macrophages (NSD)	The infiltration of macrophages into the urinary bladder suggested to reflect inflammation; as macrophage prevalence appeared to reduce (NSD), inflammation was not reported
Lombardo, Obradovic [24]	USA	Mice (C57BL/6 J)	4 to 6weeks	Female	External beam radiation	Single 15 Gy dose	Increased immunostimulatory macrophage marker CD86 observed in immunohistochemistry (NSD)	Radiotherapy appeared to decrease CD4 + T-cells, but not CD8 + T-cells (NSD)
Podmolíková, Mukanyangezi [19]	Sweden	Rats (Sprague–Dawley)	12 weeks	Female	6MV linear accelerator, administered in two side-fields	Single 20 Gy dose	Mast cells decrease (NSD) in the submucosa, increase in the detrusor muscle (NSD) and no change in the adventitia (NSD). No change in mast cell prevalence when exposed to irradiation (NSD) Marginal increase in CD206 + macrophages in the urothelium (NSD)	CD3 + lymphocytes decrease in the urothelium and lamina propria (NSD) Fibrotic area and collagen deposition significantly increased
Chen, Chen [22]	Taiwan	Rats (Sprague–Dawley)	Adult	Male	Brachytherapy targeted towards the bladder surface over the skin	300 cGy twice, over a 4 h time interval	Mast cells observed to infiltrate the urinary bladder by day 28 after chronic cystitis induction (*p < *0.05)	Interleukin (IL)-6 and tumour necrosis factor (TNF)-α were significantly higher in chronic cystitis groups
Zwaans, Krueger [8]	USA	Mice (C3H/HeN)	8 weeks	Female	Small animal radiation research platform (SARRP)	Single 20 Gy dose	No significant influx of mast cells noted in response to radiation	Mild increase in inflammatory cells (NSD) was detected in response to irradiation Irradiation caused a modest increase in fibrosis in the bladder in response to control
Giglio, Wasén [20]	Sweden	Rats (Sprague–Dawley)	Adult	Female	6MV linear accelerator, targeting the urinary bladder	Single 20 Gy dose	After irradiation, the number of mast cells decreased significantly in the urothelium, but not the detrusor muscle	The number of lymphocytes in the bladder wall was low and appeared to not be affected by radiation No differences in the expression of IL-1b, IL-4, IL-13 and IL-5 and IFN-c was observed between control and irradiated samples after 14 days. IL-1b and IL-13 expressions in the urothelium decreased in response to bladder irradiation; indicated consistent with signs of an anti-inflammatory response
Barcellos, Costa [23]	Brazil	Rats (wistar)	3–4 months	Male	10 MeV photon beam generated by a linear accelerator	2.4 Gy/min for 4.16 min to a total dose of 10 Gy	Mast cell density in the lamina propria was reduced at seven (mean 1.4; full range 0–3) and 15 (1.0; 0–2) days after irradiation compared with controls (5.2; 0–9)	

Ito, Yamamoto [21] and Lombardo, Obradovic [24] included the use of additional treatments, namely that the urinary bladders were also exposed to various forms of bladder cancer. Data have been extracted from irradiated urinary bladders without exposure to cancer or additional therapeutics. Podmolíková, Mukanyangezi [19] was contacted to specify an age for their rodent population. Barcellos, Costa [23] did not assess any secondary outcomes.

All studies utilised EBRT to irradiate the urinary bladder. The highest total radiation dose delivered to the urinary bladder was 40 Gy [21]. Three studies delivered a single dose of 20 Gy radiation to the urinary bladder [8, 19, 20], one used 15 Gy [24] and another used 10 Gy [23]. The lowest total radiation dose to the rodent urinary bladder was 300 cGy (3 Gy), delivered twice.

Five of the seven studies analyzed mast cells, and three analyzed macrophages in the irradiated urinary bladder (Table 1). In relation to mast cells, three studies assessed prevalence [8, 19, 22], and three studies assessed distribution across the urinary bladder wall [19, 20, 23]. Three studies assessed macrophage prevalence in the irradiated urinary bladder [19, 21, 24], but not distribution. One study suggested a function for macrophages in the irradiated bladder but did not directly assess macrophage function [21]. No studies examined the effect of radiation on monocytes, neutrophils, eosinophils, or basophils in the irradiated urinary bladder (Table 1).

### Effects of radiation on mast cells and macrophages in the urinary bladder

Podmolíková, Mukanyangezi [19] and Zwaans, Krueger [8] delivered a single dose of 20 Gy to the urinary bladder by external beam radiation technologies (linear accelerator or SAARP) in female Sprague–Dawley rats after two weeks and female C3H/HeN mice after 25 weeks, respectively. Both studies concluded no statistically significant difference (NSD) in total mast cell prevalence in the irradiated urinary bladder. Chen, Chen [22] also reported mast cell prevalence in Sprague–Dawley rats, finding that 28 days post-irradiation, mast cells infiltrated the male urinary bladder (*p < *0.05).

Regarding mast cell distribution, Podmolíková, Mukanyangezi [19] reported no statistical difference in mast cell number in the lamina propria (NSD), detrusor muscle (NSD) and adventitia of the urinary bladder (NSD). Barcellos, Costa [23] and Giglio, Wasén [20] reiterated these findings in male Wistar rats exposed to 10 Gy and female Sprague–Dawley rats exposed to a single dose of 20 Gy, respectively. Barcellos, Costa [23] reported no significant changes in mast cell density in the lamina propria at seven- and 15-day time points following radiation exposure (NSD) compared with controls. Similarly, Giglio, Wasén [20] reported a significant decrease of mast cells in the urothelium (*p < *0.05) but no observable changes in the detrusor muscle of the irradiated urinary bladder after 14 days (NSD).

Ito, Yamamoto [21] exposed 40 Gy of radiation to the urinary bladder of female Fischer F344 rats, finding that the prevalence of CD68þ^+^ and CD163^+^ macrophages did not change (NSD) seven days following irradiation. At 20 Gy, Podmolíková, Mukanyangezi [19] reported no changes in CD206^+^ macrophages in the irradiated urothelium (NSD). Additionally, Lombardo, Obradovic [24] observed no changes in the CD86^+^ macrophage marker (NSD).

### Inflammation, fibrosis and immunosuppression following radiation exposure

Four studies assessed inflammation in the urinary bladder following radiation exposure [8, 20–22]. Giglio, Wasén [20] reported that mRNA expressions of inflammatory markers: IL-1β, IL-4, IL-5 and IL-15 in irradiated urinary bladders were similar to that of control mRNA levels 14 days post-irradiation, suggesting that inflammatory protein expression did not change as a result of radiation exposure. Dissimilarly, semi-quantitative data analysis indicated that IL-1β and IL-13 expression was down-regulated 14 days following irradiation exposure compared to controls (NSD) [20]. Zwaans, Krueger [8] also assessed inflammation by assessing the prevalence of polymorphonuclear cells, lymphocytes, plasma cells and macrophages. A mild increase in these inflammatory cells was reported in response to a single dose of 20 Gy radiation, but was ultimately not statistically significant. Similarly, Ito, Yamamoto [21] assessed inflammation by the infiltration of macrophages into the urinary bladder, in which no changes were observed in macrophages seven days following irradiation (NSD). Supporting both Zwaans, Krueger [8] and Ito, Yamamoto [21], an inflammatory response was also reported by Chen, Chen [22] in the urinary bladders of male rats exposed to 300 cGy twice. In this study, Chen, Chen [22] reported an increase in IL-6 and TNF-α inflammatory markers in irradiated urinary bladders compared to controls, suggesting an inflammatory response.

Two studies assessed fibrosis in the urinary bladder following radiation exposure [8, 19]. Zwaans, Krueger [8] reported a modest increase in fibrosis in the urinary bladder of C3H/HeN mice in response to 20 Gy radiation, but was not statistically significant. In contrast, Podmolíková, Mukanyangezi [19] reported a significant increase in the total fibrotic area and collagen deposition of the rat urinary bladder in response to 20 Gy irradiation (*p < *0.05).

Three of the seven included studies reported on other immune cells’ prevalence in the urinary bladder [19, 20, 24]. Studies conducted by Podmolíková, Mukanyangezi [19] and Lombardo, Obradovic [24] both reported a decrease in lymphocytes in the urinary bladder. Specifically, Podmolíková, Mukanyangezi [19] reported a decrease in CD3^+^ lymphocytes in the urothelium and lamina propria of the irradiated urinary bladder; however, no statistical significance was observed. Lombardo, Obradovic [24] reported a decrease in the number of CD4^+^ T-cells (*p < *0.05) but no change to CD8^+^ T-cells in response to irradiation (NSD). Giglio, Wasén [20] reported that the prevalence of lymphocytes in the irradiated bladder was not affected by irradiation, where no statistical significance was found between control and irradiated groups.

## Discussion

Radiation-induced cystitis, a known side effect of radiation exposure to the urinary bladder, can significantly impact patient outcomes [2, 25, 26]. The lack of evidence-based research and therapeutics available in treating this disease remains a significant issue that needs to be addressed. Current pre-clinical research has demonstrated a potential role for macrophages [6] and mast cells [7–11] in the pathophysiology of radiation-induced toxicities; however, a clear link has not been established for the involvement of these immune cells, and other myeloid-derived immune cells, in the prognosis of radiation-induced cystitis.

This systematic review revealed that the effect of radiation on myeloid-derived immune cells in radiation-induced cystitis is poorly defined. In other literature, a role for the urinary bladder mast cell [3, 5] in the pathophysiology of radiation-induced cystitis has been suggested. In the healthy urinary bladder, mast cells are critical regulators of immunity [27–29] distributed throughout all layers of the urinary bladder wall [30]. In the context of radiation-induced toxicities, mast cells have been associated with the development of radiation-induced fibrosis around various tissues of the body [7–11]. Specifically, mast cells are believed to release various chemokines, such as TGF-β, which cause fibrosis by promoting fibroblast recruitment and proliferation [12]. Mast cells are also believed to disrupt vasculature by increasing the permeability of blood vessels [16] as well as playing a direct role in chemical cascades that result in radiation-induced inflammation [9].

In the reviewed studies, several investigated mast cells in the irradiated urinary bladder [8, 19, 20] [22, 23]. Two studies indicated no statistical difference in the infiltration of mast cells between irradiated and non-irradiated groups [8, 19]. These studies suggest that mast cells are resistant to radiation exposure in the urinary bladder, consistent with reports on mast cells exhibiting resistance to radiation-induced cytotoxicity [31, 32]. Contrastingly, two studies indicated a decrease [20, 23] and one study indicated an increase in mast cell density following irradiation [22]. Mast cells were reported to decrease in the urothelium [20] and lamina propria [23], which may suggest that mast cells are more susceptible to radiation-induced toxicities in the most apical layers of the urinary bladder wall following irradiation at 14-day, and 7- and 15-day timepoints, respectively. After 28 days, Chen, Chen [22] reported that mast cells infiltrated the bladder, suggesting that this increase in mast cells may be indicative of inflammatory response. Despite this, further research is needed to validate these findings.

In addition to mast cells, macrophages have also been implicated in the development of radiation-induced toxicities through similar mechanisms of inflammation and fibrosis [33]. Macrophages were investigated in three of the included studies [19, 21, 24], in which all three studies concluded no statistical difference in macrophage prevalence following irradiation. Despite no statistical difference between non-irradiated and irradiated groups, Podmolíková, Mukanyangezi [19] and Lombardo, Obradovic [24] reported a marginal increase in CD206^+^ macrophages and increased CD86^+^ macrophage-associated markers in the urinary bladder. Ito, Yamamoto [21], however, reported a reduction in CD68þ macrophages, suggesting that CD68þ macrophages may be susceptible to radiation.

Though previous research has suggested a role for urinary bladder mast cells and macrophages in the pathophysiology of radiation-induced cystitis, this systematic review has determined that these cells do not have a clear prognostic value in the development of this disease. Firstly, only four studies assessed inflammation following irradiation of the urinary bladder [8, 20–22], two of which identified inflammation in the irradiated urinary bladder [8, 20]. Incongruencies in techniques used to assess cystitis, as well as a lack of assessment in several studies, make it difficult to draw meaningful conclusions about the involvement of mast cells or macrophages in relation to radiation-induced cystitis, a disease characterized by inflammation. Thus, it is recommended that a guideline is developed for the assessment of radiation-induced cystitis in pre-clinical models to allow for more meaningful and accurate comparisons in the assessment of radiation-induced cystitis, with particular regard to its triphasic development [3, 4].

Of particular interest was the lack of any literature on the impact of myeloablative radiotherapies, or the associated HSCT on myeloid lineage immune cells. Despite its curative potential, HSCT and its conditioning regiments have been linked to radiation-induced cystitis. The development of radiation-induced cystitis following HSCT is associated with increased disease morbidity, prolonged hospitalisations [2, 25] and significant increases in non-relapse-related mortality, according to Galli, Sorà [26]. The prevalence of radiation-induced cystitis, associated with HSCT conditioning regimens, has a reported prevalence ranging between 10% and 20% in paediatric transplant recipients [34] and 17% in adult transplant recipients [35], with the median incidence of cystitis reported to occur within the first 30 days of transplantation [26]. Due to its prevalence and apparent risk to immunocompromised patients, future research on HSCT and myeloablative radiotherapies is highly recommended.

Only three of the included studies in this systematic review assessed the prevalence of other immune cells in the urinary bladder [19, 20, 24], and found that there were no statistically significant differences in assessed lymphocytes [19, 20]. However, Lombardo, Obradovic [24] reported no change to the number of CD4^+^ and CD8^+^ T-cells in response to irradiation compared to controls (NSD). To this end, it is well known that myeloablative radiotherapy can induce immunosuppression [36]. As all included studies utilized EBRT to evaluate myeloid-lineage immune cells in the irradiated urinary bladder, it is unclear as to whether these findings are applicable to myeloablative radiotherapy. Dually, it is also recommended that future research is done to validate a model of radiation-induced cystitis, in consideration of different species, strains, dosimetry, and modes of radiation. Due to scarcity in the assessment of other myeloid-lineage immune cells, namely monocytes, neutrophils, eosinophils and basophils, future studies should also aim to assess the function, prevalence, and distribution of a variety of immune cells in the irradiated bladder, focusing on how these characteristics may contribute to disease pathology or how they may differ in the context of myeloablative radiotherapy.

Limitations of this systematic review include the small number of identified studies and the inability to make valuable comparisons due to the different species, strains, dosimetry, and techniques used. Additionally, it is important to recognise that only four of the included studies sought to investigate cystitis resulting from radiation exposure [8, 19, 20, 22]. The remaining three studies [21, 23, 24] had discussed inflammation resulting from radiation exposure, but did not explicitly identify cystitis. These studies were still included in this systematic review, however, as they still met the inclusion criteria.

## Conclusion

In summary, this systematic review highlights a need for further research into the pathology of radiation-induced cystitis. Further research is needed to identify the role of myeloid-lineage immune cells in the development of radiation-induced cystitis (amongst other radiation-induced toxicities in the urinary bladder), particularly in the context of myeloablative radiotherapy. Specifically, pre-clinical research should further investigate the potential prognostic role of mast cells, macrophages, and other myeloid-lineage immune cells in the development of radiation-induced cystitis, as well as continuing to refine current models of radiation-induced cystitis to aptly reflect the pathophysiology of this disease.

## Data Availability

Data can be made available upon reasonable request by emailing the corresponding author.
